# Adaptive Decision‐Making “Fast” and “Slow”: A Model of Creative Thinking

**DOI:** 10.1111/ejn.70024

**Published:** 2025-03-10

**Authors:** Radwa Khalil, Martin Brüne

**Affiliations:** ^1^ School of Business, Social, and Decision Sciences Constructor University Bremen Germany; ^2^ LWL University Hospital Bochum, Department of Psychiatry, Psychotherapy and Preventive Medicine, Division of Social Neuropsychiatry and Evolutionary Medicine, Ruhr University Bochum Bochum North Rhine‐Westphalia Germany

**Keywords:** adaptive decision‐making, creativity, fast and slow thinking, heuristics and biases, novelty, uncertainty

## Abstract

The late Daniel Kahneman introduced the concept of *fast and slow thinking*, representing two distinct cognitive systems involved in decision‐making (DM). Fast thinking (System 1) operates intuitively and spontaneously. In contrast, slow thinking (System 2) is characterized by deliberation and analytical reasoning. Following Kahneman's view, called *the biases* view, we suggest a framework involving the interplay between two systems, the bottom‐up and top‐down approaches. These two approaches involve various modalities, including learning skills, perception, cognition, attention, and emotion. Accordingly, we incorporate temporal modulation, which varies based on individual differences and accounts for adaptive DM. Our overarching framework elucidates how the brain dynamically allocates resources for adaptive DM and how creative mental processes could drive it. We highlight the immense value of interdisciplinary research collaboration in progressing the empirical research of our proposed framework.

## Graphical abstract

The adaptive decision‐making model incorporates a search type involving dual clustering and switching systems. It also integrates fast and slow strategies and temporal modulation, which varies based on individual differences. The brain model depicts a dual system of bottom‐up and top‐down processes, illustrated by the two arrows. These processes involve interaction between various modalities, including learning skills, perception, cognition, attention, and emotion.

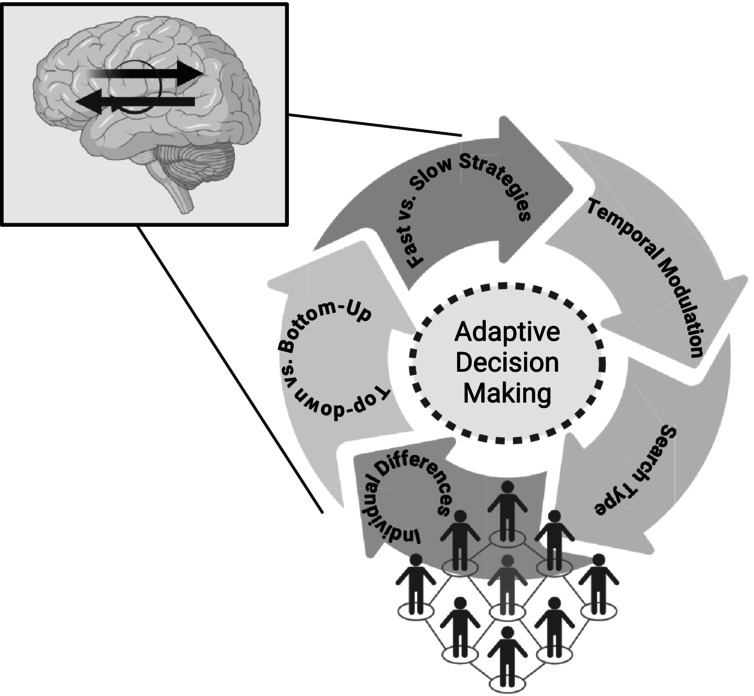

**Abbreviations**
BR: bounded rationality
DM: decision‐making
JDM: judgment and decision‐making
LHS: life history strategies

## 1 Introduction

*Thinking Fast and Slow*, published by the late Kahneman (2011), deals with the distinction of two cognitive systems involved in decision‐making (DM). The fast thinking (System 1) functions intuitively and spontaneously, while slow thinking (System 2) is characterized by cautious and analytical reasoning. However, human DM often relies on heuristics and biases, leading to erroneous judgments and decisions, particularly in uncertain situations (Gilovich and Kahneman 2002; Kahneman and Tversky 1984; Tversky and Kahneman 1974). *Uncertainty* is defined as an organism's limitation(s) to accurately assess the likelihood of a future environmental event(s) (Downey and Slocum 1975). It is related to a lack of knowledge or predictability about outcomes and encompasses the variability and unpredictability inherent in DM situations. In judgment and decision‐making (JDM), a primary focus is how individuals evaluate risks and make choices (Scott 1993). The interplay between *uncertainty* and *rationality* has been a long‐standing research subject across disciplines, including economics, philosophy, psychology, and ecology (Downey and Slocum 1975; Milliken 1987). Traditional models emphasize rational optimization in the face of *uncertainty*. In economics, the premise of global rationality is replaced by rational conduct. This economic model indicates the capacity of species (including humans) to gather information^1^ and use their computational skills within the ecosystems they inhabit (Simon 1955).

Individuals converge sensory information and prior knowledge to form beliefs or predictions about future events even when unaware of the connections between their actions and the surrounding environmental factors (Downey and Slocum 1975). The relationship between *uncertainty* and *rationality* in ecological contexts is linked to ecological rationality, indicating that environmental uncertainties influence DM. This perspective is based on Herbert Simon's concept of bounded rationality (BR) (Simon 1957) and Egon Brunswik's lens model (Brunswik 1955).^2^ Simon's BR occupies a somewhat intermediate position, offering an alternative perspective on *Homo economicus*.

At the heart of the ongoing discussion surrounding DM lies the interconnectedness of rationality and heuristics. Heuristics often serve as a practical means to achieve *rationality* within the constraints of human cognition (Gigerenzer and Goldstein 1996; Gilovich and Kahneman 2002; Kahneman and Tversky 1984; Tversky and Kahneman 1974). Two primary viewpoints on heuristics emerge: the biases proposed by Kahneman and Tversky (Kahneman and Tversky 1984; Tversky and Kahneman 1974) and Gigerenzer's fast and frugal model (Gigerenzer and Goldstein 1996). The central disagreement between these viewpoints is based on the appropriate normative standard for assessing human behavior (Vranas 2000). Gigerenzer contends that the heuristics and biases perspective inaccurately categorizes all biases as errors, particularly those that can be accurate and beneficial in DM scenarios (Gigerenzer and Brighton 2009). Gigerenzer's method^3^ questioned the assumption that heuristics are invariably nondeliberate cognitive processes, which has fueled an ongoing intellectual debate within economics. While the Gigerenzer method argues the validity of the dual‐system model in DM (Kruglanski and Martignon 2011), Kahneman and Tversky argue that the dispute is just a matter of terminology (Gigerenzer 1996; Kahneman and Tversky 1996).

Kahneman's influential biases view has sparked considerable debates in contemporary cognitive psychology. However, it is crucial to recognize that dual‐process theory (DPT) predates Kahneman's groundbreaking contributions, with notable work from scholars such as Epstein, Sloman, and Stanovich. These scholars have examined similar cognitive divisions since the 1990s (Sloman 1996; Stanovich and West 1998). Sloman (1996) investigated DPT by distinguishing between associative and rule‐based reasoning, which complicates the simplistic fast–slow dichotomy by illustrating that rule‐based systems can inhibit associative processes but do not entirely suppress them. Evans (2008) and Evans and Stanovich (2013) emphasized the complexities of cognitive systems, supporting a focus on the quantitative differences between processes rather than strictly adhering to a binary classification. Recent critiques of DPT stress the necessity of moving beyond the oversimplified System 1/System 2 framework. Relational knowledge plays a significant role in influencing cognitive processes, suggesting that fast, automatic responses are not solely under the control of System 1 (De Houwer 2019). This perspective is supported by findings indicating that cognitive processes are more interconnected than the DPT proposes, thereby questioning the notion that these systems operate independently (Kruglanski and Martignon 2011).

The implication of DM as a trade‐off between effort and accuracy is widely established. Simon (1957) noted that individuals often experience satisfaction even when they cannot fully maximize their potential. Due to the substantial costs associated with implementing the principles of global rationality (Good 1952), individuals tend to prioritize cognitive efficiency over accuracy (Payne, Bettman, and Johnson 1988). Therefore, the assumption that the accuracy‐effort trade‐off is universal—a common argument employed to support heuristics as valid models for DM—is questionable (Hogarth 2012; Katsikopoulos 2010). Given individuals' cognitive limitations, BR provides a more realistic perspective on *rationality* (Klaes and Sent 2005; Simon 1957). Behavioral economics is typically associated with Kahneman's heuristics and biases (1974). However, it often overlooks evolved cognitive and emotional constraints based on kin selection, such as moral conduct, reciprocal altruism, and altruistic (costly) punishment, which are at odds with rational DM (Trivers 1971). Consequently, *rationality* can be viewed from various angles. (1) Procedural rationality, developed by Simon (1976), evaluates the trade‐off between DM costs and quality. (2) Ecological rationality focuses on the relationship between global environmental factors and DM. Gigerenzer's^4^ concept of ecological rationality offers an influential contemporary method for adaptive DM. In their research, Gigerenzer and Brighton introduced the bias‐variance dilemma to demonstrate how the human mind benefits from utilizing a diverse set of specialized heuristics and biases, efficiently creating an adaptive toolbox (Gigerenzer 2001; Gigerenzer and Brighton 2009; Pachur et al. 2012). According to the bias–variance trade‐off, the ability to make accurate predictions indicates that errors are primarily driven by variance when operating with limited data. Nevertheless, our cognitive system often manages these errors efficiently, achieving acceptable accuracy (Gigerenzer and Brighton 2009).

Novel cues are intrinsically associated with *uncertainty* (Carscadden, Batstone, and Hauser 2023). Research on *uncertainty* has explored neophobia^5^, the fear of new experiences, and memory attenuation (Carscadden, Batstone, and Hauser 2023). Studies have revealed that when exposed to unpredictable predator diversity. Animals like tadpoles and guppies exhibit neophobia in response to a novel odor; however, those exposed to low *uncertainty* did not display such a reaction (Feyten et al. 2019). Generalization occurs when an unfamiliar cue resembles a familiar one. This process allows individuals to apply knowledge from known to unknown inputs. In situations of *uncertainty*, the process of learning and retention of new responses may be prolonged, indicating that higher levels of *uncertainty* can immediately impact behavior (e.g., response retention or memory).

Small samples^6^ suggest that our short‐term memory constraints may be an adaptation to the environment we encounter throughout our lives. Consequently, *novelty* can be viewed as an opportunity; new situations often stimulate exploration, prompting the search for new approaches or solutions. In DM, *novelty* can serve as a chance to acquire knowledge, adapt strategies, and mitigate the *uncertainty* that arises from ambiguity (Nadel and Willner 1980). When faced with environmental novelty, individuals may adopt exploratory DM strategies. In this case, individuals actively seek new information and remain open to alternative solutions; thus, *novelty* befalls a catalyst for learning (i.e., learning orientation). Often, organisms repeatedly encounter a cue multiple times to fully understand its associated information and contextual dependencies.

In some cases, gathering additional cues may not reduce *uncertainty*; however, cue overload necessitates integrating and sorting the available information (Munoz and Blumstein 2012). Consequently, incomplete cues can hinder the perception of risk related to sensory modalities^7^ (Crane, Achtymichuk, et al. 2023; Crane, Feyten, et al. 2023). Crane and colleagues concluded that such DM rules involve manipulating past experiences with threatening and nonthreatening cues at varying frequencies. Thus, if we can evaluate the reliability of these cues, we should implement a decision rule that assigns weights in proportion to their reliability. Multiple novel stimuli can lead to cognitive overload and *uncertainty*, complicating information processing.

The explore–exploit dilemma is a central theme in DM and foraging literature (Costa, Mitz, and Averbeck 2019; Lin 2013; Todd and Hills 2020; Wilson et al. 2014). By framing novelty‐seeking within this trade‐off, we can draw parallels between broader DM scenarios and foraging choices. Wilson et al. (2014) emphasized how humans utilize directed and random exploration to navigate DM dilemmas, suggesting that novelty‐seeking behaviors can influence choices even in more static contexts. Likewise, Gottlieb et al. (2013) emphasized the role of curiosity in driving exploratory behavior and DM across various contexts. The ability to flexibly switch between exploration and exploitation and between inhibition and disinhibition during novelty‐seeking tasks is essential for characterizing creative abilities (Ivancovsky, Baror, and Bar 2024). This perspective may provide a theoretical foundation on how *novelty* affects decisions beyond traditional foraging scenarios, i.e., creative DM. Traditional literature on general DM (i.e., JDM) is often framed around rationality and optimality, which may only partially capture the complexities of foraging behavior. Researchers such as Kahneman and Gigerenzer have critiqued the focus on optimality, advocating for a more realistic understanding of how people make choices based on BR and heuristics (Sloman 1996; Stanovich and West 1998).

Adaptation and optimality are frequently regarded as contrasting concepts in the JDM literature. Adaptation generally refers to modifying behaviors and decisions to effectively address situational demands rather than striving for an ideal performance standard. This perspective is supported by economic models, such as the one proposed by Stigler (1961). This model equates adaptation with optimality but predates the critical debates initiated by Simon, Kahneman, and Gigerenzer. Adaptation is usually understood as pursuing a “good enough” solution instead of an optimal one, which aligns with Simon's concept of satisfaction. This concept posits that individuals often seek satisfactory solutions rather than optimal ones when confronted with complex decisions (Evans 2008). In evolutionary biology, the term “optimal adaptation” is widely used, which asserts that organisms evolve traits that maximize their fitness within a specific environment. This term is misleading because design optimality does not exist in nature. Similarly, human DM often diverges from these optimal benchmarks due to cognitive biases and BR. Kahneman's research on heuristics and biases demonstrates that individuals frequently employ mental shortcuts that result in systematic errors rather than optimal choices (Sloman 1996). Gigerenzer argues for fast and frugal heuristics, which are adaptive but not necessarily optimal in the conventional sense (Stanovich and West 1998). Semantic foraging^8^ has significant implications for the frameworks used to analyze DM processes (Abbott, Austerweil, and Griffiths 2015; Hills, Jones, and Todd 2012; Mobbs et al. 2018; Todd and Hills 2020). Pirolli's work on information foraging theory emphasizes how individuals navigate information environments to maximize their knowledge gain (Pirolli 2005). By focusing on semantic foraging, we can utilize extensive literature that examines how cognitive processes, environmental cues, and individual differences influence DM in foraging contexts. Optimal foraging theory^9^ posits that organisms develop strategies to maximize their energy intake relative to the time and energy spent foraging (Hills, Jones, and Todd 2012).

The following sections discuss conceptual similarities between Kahneman's dichotomy of fast and slow cognitive processes (Kahneman 2011), novelty emergence, and adaptive DM. This discussion underlines the connection between fast and slow cognitive strategies, creative thinking, and adaptive DM. We propose a dual system that should consider bottom‐up and top‐down processes to view adaptive DM processes as an analog of fast and slow thinking modes. We discuss how an evolutionary framework for adaptive DM can be valuable for understanding this process across various species. Finally, we suggest bridging the segregated literature from diverse disciplines and incorporating brain and cognitive science literature into a holistic framework to advance research on adaptive DM.

## 2 Fast and Slow Cognitive Strategies and Creative Thinking: Is There a Tie?

Ecologically relevant environmental novelty interferes with *thinking fast and slow*, moving beyond established patterns and potentially hindering the generation of novel ideas. This interruption may be constrained by heuristics and biases (Gilovich and Kahneman 2002; Kahneman and Tversky 1984; Tversky and Kahneman 1974). The roots of *novelty* are profoundly intertwined with the notions of natural selection and genetic variation, which contribute to the diversity and adaptation of living organisms. Müller and Wagner (1991) indicated that *novelty* cannot arise from losing a gene or trait; however, many scholars disagree. Focusing solely on adaptive radiation overlooks many novel features that emerged before ecological opportunities for niche differentiation and radiation (Erwin 2015). Some researchers assert that new phenotypes resulting from gene loss (Ochman and Moran 2001), new combinations of existing traits, and changes in the number of traits—such as hybrid offspring being larger than either parent species—are also forms of *novelty* (Pigliucci 2008). The most inclusive definition contends that any character or variation should be considered a *novelty*, regardless of its significance (Arthur 2000). The definition of *novelty* in the context of fast and slow thinking can encompass (1) an inherent trait of an organism, (2) a characteristic feature of the environment, or (3) embracing *novelty* that emerges from a creative mental process. Since organisms do not live in isolation, their interactions with the environment can also give rise to novel forms. Therefore, we must clarify our definitions of novelty to develop comprehensive and coherent literature that reveals common processes underpinning varied novelty forms. Until now, our knowledge of ecological novelty often focuses on novel cues or occurrences rather than biological novelty.^10^ It is essential to discriminate between various forms of novelty, i.e., environmental or ecological novelty and biological (trait) novelty. Numerous conflicting approaches to defining *novelty* exist, supporting the necessity for an interdisciplinary approach to comprehending the role of ecological novelty in DM.

From an evolutionary point of view, both fast and slow thinking can have benefits or downsides concerning survival and reproduction. While fast DM can be advantageous in uncertain environments, it may be suboptimal in more complex situations where slow analytical thinking pays off (Gilovich and Kahneman 2002; Scott 1993; Tversky and Kahneman 1974). Evolutionary biases may have served as cognitive shortcuts, enabling our ancestors to make quick decisions with limited information in specific situations (Nowak and May 1992). Access to more information can assist individuals in avoiding relying on heuristics based on past experiences when making inferences. Moreover, it is crucial to consider the other side of the coin by viewing human societies as an environment of adaptive values that influence the evaluation of individual behavior (Boyd and Richerson 2005). Imitation is a prevalent tactic in humans, even observed in preverbal newborns (Gergely, Bekkering, and Király 2002). This tendency plays a significant role in cultural transmission (Boyd and Richerson 2005) and the evolution of social norms (Bicchieri and Ryan 2014). Only a few individuals are committed to improving cultural practices, such as folklore, to promote cultural adaptation. As a result, human societies have an advantage over other social animals, as cultural adaptation occurs significantly faster than genetic adaptation (Bowles and Gintis 2011).

Animal studies may go beyond what we know about the learning process, problem‐solving abilities, cognitive flexibility, and novelty seeking (Griebel and Kimbrough Oller 2008; Kaufman and Kaufman 2015; Ramsey, Bastian, and van Schaik 2007). Bower birds exhibit aesthetic sensitivities, evidently echoed in the architectural design of their bowers (Ramsey, Bastian, and van Schaik 2007). Songbirds have demonstrated remarkable flexibility and elaborateness in their song production (Griebel and Kimbrough Oller 2008). Disneynature's 2012 documentary “Chimpanzee” offers a compelling example of nonconformist behavior. In this film, an alpha male chimpanzee, unrelated to an infant chimp, assumes a caregiving role after the infant's mother has died. These examples emphasize the potential of animals to engage in novelty‐seeking and utilize emotion‐driven DM, even when the resulting behavior comes at significant costs (Wiggins et al. 2015).

Another intriguing phenomenon related to creativity is synesthesia, which can be classified as cross‐modal or unimodal (Cytowic 2002; Mitchell 2010). Hubbard and Ramachandran (2003, 2005) defined *synesthesia* as an automatic, consistent, and conscious experience. In cross‐modal synesthesia, the stimulation of one sensory system can evoke perceptions in another modality. For instance, the sound of a church bell might trigger a visual experience of various colors. Conversely, unimodal synesthesia involves stimulation within a single sensory modality that leads to multiple characteristics within that same modality. Nevertheless, synesthetic sensations are highly constant, limiting association evolution. If a person consistently perceives the color “pink” when encountering the number “5”, this reflects a restricted range of associations. The capacity to connect seemingly unrelated ideas can enhance creativity, as creative thinking often hinges on forming associations between disparate concepts (Beaty and Kenett 2023; Checiu, Bode, and Khalil 2024; Kenett 2024; Kenett and Faust 2019; Mtenga, Bode, and Khalil 2024). Creative thinking is a dynamic state of mind that requires cognitive flexibility for its cultivation and growth (Khalil et al. 2023; Wilson, Guilford, and Christensen 1953; Zmigrod, Zmigrod, and Hommel 2015). The dynamic nature of creative thinking (Agnoli 2024; Corazza, Agnoli, and Mastria 2022) is expressed in various forms, colors, and shapes, making it a complex and intellectually inspiring area of study (Abraham 2024; Khalil and Demarin 2024; Khalil and Moustafa 2022). By examining the components of creative mental processes—such as cognitive flexibility—we can simplify this complexity and explain how these elements interact.

## 3 How Do Creative Mental Operations Influence the Trade‐Off Between Fast and Slow Thinking?

Our perspective emphasizes the value of adapting to novel circumstances in DM. Efficiently managing *uncertainty* by responding to relevant changes and implementing successful strategies is essential for achieving an optimal level of adaptive DM. Recently, Nussenbaum et al. (2023) underlined that *novelty* can alleviate the adverse effects of *uncertainty*, suggesting that the availability of new options may prompt exploration even in uncertain environments. Consequently, *novelty* acts as a motivational factor that drives DM processes. Cockburn et al. (2022) provide evidence that *novelty* and *uncertainty* modulate the balance between exploration and exploitation through distinctive mechanisms within the human brain.

**FIGURE 1:**
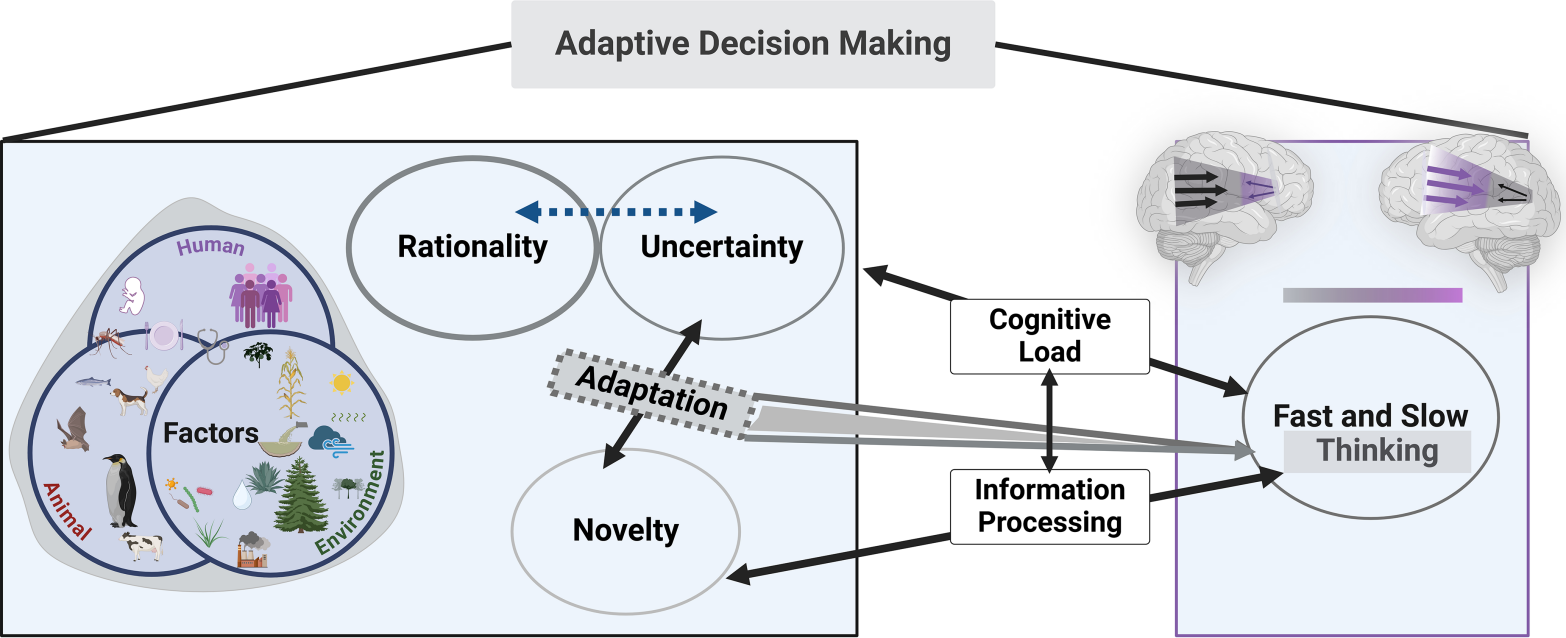
Abstract framework for adaptive decision‐making (DM). Adaptive behavior in DM can elucidate the connection between rationality, uncertainty, novelty, and the interplay of fast and slow thinking. An abundance of novel stimuli in uncertain contexts may result in cognitive overload, affecting information processing. Individuals engage in adaptive DM, influenced by various external factors, including the physical environment surrounding humans and animals. The blue dashed arrow between rationality and uncertainty signifies the differing perspectives of behavioral economics and ecological disciplines. Novelty fosters exploration and learning, while uncertainty increases the demand for additional information; nevertheless, novelty and uncertainty are interconnected through adaptation.

New information and circumstances generally refer to any changes in the external environment or internal state that require a response (Crane, Achtymichuk, et al. 2023). These changes may include novel situations, unexpected events, or shifts in the context that necessitate reevaluating existing knowledge or strategies (Anderson 1991; Laureiro‐Martínez and Brusoni 2018; Nadel and Willner 1980). Adaptability is a broad concept that encompasses the ability to respond to new information and integrate past experiences, which leads to anticipating future challenges and innovating in unfamiliar situations. It is indispensable to recognize that *uncertainty* is not inherently linked to *novelty*. While *uncertainty* often prompts individuals to seek additional information, *novelty* encourages exploration, learning, and adaptability. Hence, achieving a nearly optimal level of adaptive DM may involve finding a balance between navigating *uncertainty*, responding to ecologically relevant *novelty*, and utilizing effective strategies. When individuals encounter high levels of *uncertainty*, they often resort to familiar heuristics or past experiences. Conversely, they are generally more open to exploring new options in novel situations. Thus, adaptive behavior in DM can elucidate the connection between *novelty, rationality, uncertainty,* and the interplay between fast and slow thinking. We propose a comprehensive framework for adaptive DM that integrates dual systems, incorporating bottom‐up and top‐down processes akin to fast and slow thinking modes (Figure 1).

This dual system (bottom‐up and top‐down) includes several modalities: learning skills, perception, cognition, attention, and emotion. The trade‐offs within these modalities can be described by several contrasts: spontaneous versus deliberate modes, local versus global attention, narrow versus broad cognitive considerations, and exploitative versus exploratory learning strategies. We propose that individual differences significantly affect the degree of variation observed in this trade‐off. Through this dual system, the brain functions engage in a dynamic competition between motor and nonmotor loops, which coordinate and integrate these functions (Boraud, Leblois, and Rougier 2018; Guthrie et al. 2013; Héricé et al. 2016; Topalidou et al. 2018). The interconnected loops comprise the cortex, striatum, pallium, and thalamus. Information flows from the cortex to the striatum, then to the pallium, followed by the thalamus, and ultimately returns to the cortex via the thalamus (Guthrie et al. 2013; Héricé et al. 2016; Topalidou et al. 2018). These competitive dynamics accentuate the brain's ability to adaptively allocate resources according to environmental demands to promote efficient DM processes.

Fast and slow clustering and switching processes offer valuable premises for the adaptive DM through semantic foraging (Hills, Todd, and Jones 2015). In foraging contexts, *novelty* is inherently tied to pursuing new information and resources, as organisms must continuously adapt their strategies in response to changing environmental conditions (Addicott et al. 2017; Mobbs et al. 2018). Costa et al. (2014) and Menegas et al. (2018) explore how dopamine exploits novelty‐seeking behavior during DM and approach‐retreat behaviors in foraging, emphasizing the significance of understanding the neurological mechanisms underlying these behaviors. Nevertheless, these methodological approaches, which can be interpreted as risk assessments, are less applicable to conventional JDM tasks. JDM scenarios typically focus on static choices with predefined outcomes, such as lotteries, where *novelty* is minimal. Although *novelty* plays a crucial role in foraging decisions, its significance within the broader context of general DM (i.e., JDM) may warrant greater attention.

Research on semantic foraging relies on the marginal value theorem (Charnov 1976) alongside the classical distinction between clustering and switching in fluency tasks (Troyer, Moscovitch, and Winocur 1997). Memory retrieval encompasses a blend of automatic and controlled processes, which play a role in both switching and clustering (Hills et al. 2013; Hills, Todd, and Jones 2015; Hills and Pachur 2012; Lundin et al. 2023; Rosen and Engle 1997; Troyer, Moscovitch, and Winocur 1997; Troyer et al. 1998). Creative ideas emerge from unconscious processes, such as association, spontaneity, and conscious, controlled, and goal‐oriented processes (Zioga et al. 2024). How individuals search for ideas within their memories is a fundamental component of creative thought (Todd and Hills 2020; Troyer, Moscovitch, and Winocur 1997; Troyer et al. 1998). Recent studies have explained creative ideation through memory retrieval, variations in memory structure, and the underlying brain networks involved (Beaty and Kenett 2023; Benedek et al. 2023; Kenett and Faust 2019; Mildner and Tamir 2019; Ovando‐Tellez et al. 2023). Mednick's 1962 hypothesis suggests creative individuals have flatter associative hierarchies, enabling faster connections between distant concepts. This cognitive process uses mental shortcuts during moments of insight (Schilling 2005), requiring frequent and rapid cognitive transitions, suggesting adaptable exploration approaches. Therefore, variations in the speed of these cognitive transitions—whether in response to related or unrelated associative hierarchies—may reflect semantic shortcuts tied to typical memory organization and, consequently, influence DM.

The study by Ovando Tellez et al. (2024) differentiated between two types of cognitive switching: fast and slow. Fast switches symbolize spontaneous switching and are aligned with the flexibility component of the creative thinking dual pathway model (Nijstad et al. 2010), which contrasts flexibility with persistence pathways. Slow switches indicate controlled switching (Mastria et al. 2021; Troyer, Moscovitch, and Winocur 1997). Clustering in creative ideation supports the persistence process of the dual pathway model (Nijstad et al. 2010), allowing for the exploitation of idea clusters. Switching requires a substantial investment of cognitive resources during creative ideation, often leading to longer search times (Hass 2017; Mastria et al. 2021). Thus, the transition between behaviors can occur quickly or more deliberately, i.e., slow rather than fast switching aligns with optimal foraging theory (Ovando Tellez et al. 2024). As a result, fast and slow switching involves various cognitive processes, each with varying degrees of associative thinking and cognitive control. These findings also emphasized the significant role of search type (clustering vs. switching) and its temporal modulation (fast vs. slow) in understanding individual differences in creativity‐based semantic associations. For example, fast switching between brain clusters enhances the ability to link remote associations in memory, which are tied to spontaneous brain connectivity; in contrast, slow switching impacts the relationship between brain connectivity and creative divergent thinking. Switching flexibly between categories or semantic representations may enhance an individual's capacity to connect more remote associations within memory (Bendetowicz et al. 2018; Lee and Therriault 2013).

The relationship between switching and memory‐based creative tasks is fascinating, as creative individuals typically exhibit greater cognitive flexibility and are prone to activating multiple lexico‐semantic meaning representations (Beaty and Kenett 2023; Benedek et al. 2023). The global transitions between clusters occur when the retrieval rate within a cluster falls below a specific threshold (Troyer, Moscovitch, and Winocur 1997). These are called local–global transitions and relate to exploration and exploitation processes (Charnov 1976; Hills, Todd, and Jones 2015; Hills and Kenett 2025; Hills and Pachur 2012). Search processes in creative cognition (Fauconnier and Turner 1998; Kaplan and Simon 1990; Simonton 2003; Ward and Smith 1997; Ward, Smith, and Vaid 1997), whether exploratory or exploitative, play a vital role in generating novel ideas (Hills, Todd, and Goldstone 2008). Interestingly, the organization of semantic categories (clustering) and the switching between these categories are associated with various semantic network properties and creative abilities (Bieth et al. 2024; Herault et al. 2024; Mastria et al. 2021; Ovando‐Tellez et al. 2022; Ovando Tellez et al. 2024; Troyer, Moscovitch, and Winocur 1997; Zhao, Guo, and Hong 2013).

**FIGURE 2:**
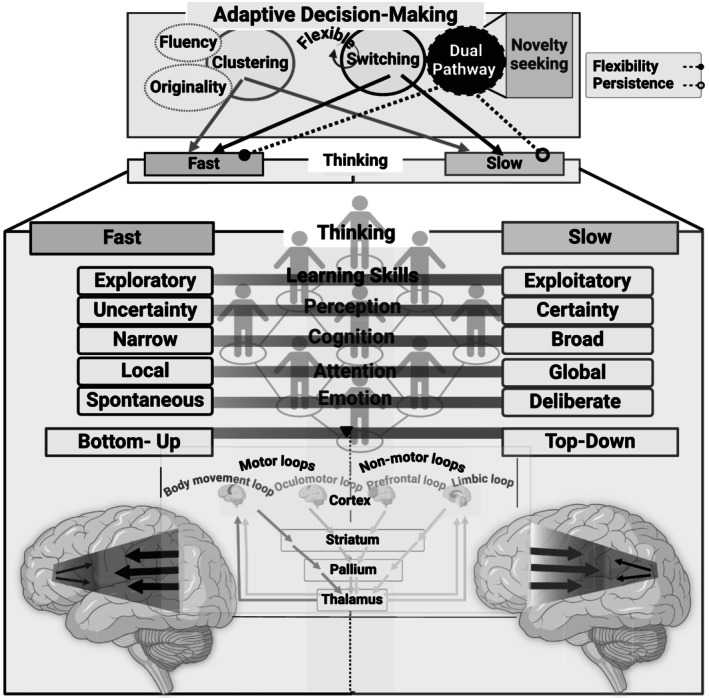
Holistic framework for adaptive decision‐making (DM) in creative thinking. This framework encompasses two systems—clustering and switching—alongside temporal modulation, which includes fast versus slow thinking and bottom‐up versus top‐down approaches that account for individual differences. Clustering is primarily associated with fluency and originality, while switching is related to dual pathways of creativity. The flexible, creative pathway leans towards fast switching, and the persistent, creative pathway favors slow switching. Novelty‐seeking is closely linked to flexible switching and dual pathways. The dual system of bottom‐up and top‐down approaches integrates multiple modalities, such as learning skills, perception, cognition, attention, and emotion. These modalities symbolize a trade‐off between spontaneous versus deliberate modes, local versus global attention, narrow versus broad cognitive thoughts, uncertainty versus certainty, and exploratory versus exploitative learning skills. These trades vary among individuals. The dual bottom‐up and top‐down processes govern dynamic competition between motor and nonmotor loops. Key brain regions, including the cortex, striatum, pallium, and thalamus, are involved in these loops. The flow of information spans from the cortex to the striatum, to the pallium, to the thalamus, and back to the cortex through the thalamus. This dynamic competition exemplifies the brain's ability to allocate resources adaptively, respond to environmental demands, and ultimately enable adaptability and efficient functioning in DM.

While many researchers argue that these switching processes are mainly controlled, other studies indicate that switching can occur spontaneously and may have an associative nature (Hills, Todd, and Jones 2015; Lundin et al. 2023). Accordingly, both systems are operating together and not separately. For instance, System 2 thinking—characterized as slow, deliberate, and analytical—plays a vital role in complementing the creative mental functions of System 1 by assessing and refining the ideas it generates. Therefore, creative thinking should not be viewed simply as a binary choice between fast and slow; instead, it exists on a continuum, switching between these two systems. Acknowledging the considerable impact of individual differences on DM can explain how individuals switch between two cognitive systems. It also explains why certain behaviors, which may initially appear ineffective, can still succeed in achieving some individuals' goals under specific circumstances. In other words, a seemingly inefficient behavior may effectively fulfill its purpose when contextual factors are considered (Anderson 1991; Marr 1982; Oaksford and Chater 1994; Palmer 1999). With this in mind, we propose a framework for adaptive DM in which the dual processing system alternates between bottom‐up and top‐down approaches. The extent of these transitions is influenced by individual differences and their responses to environmental cues (Figure 2).

**FIGURE 3:**
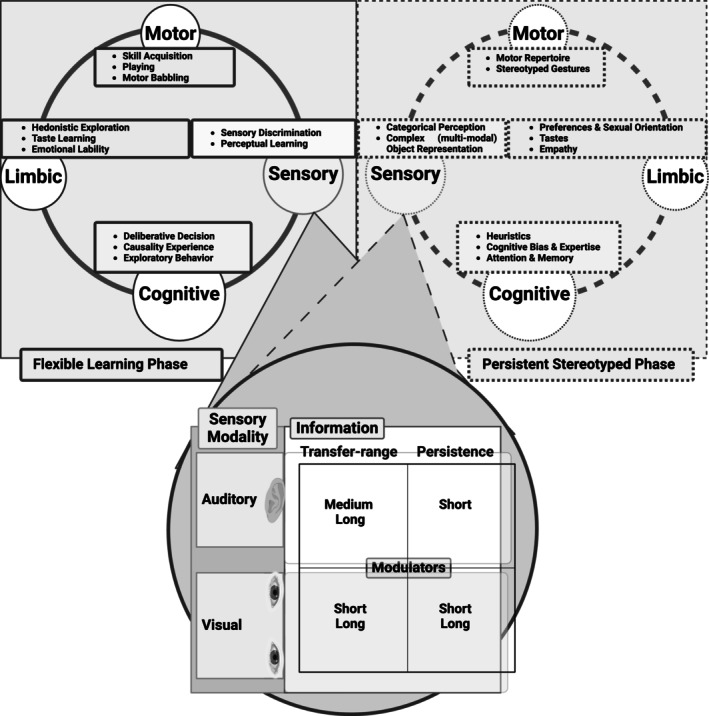
Description of adaptive decision‐making (DM) through the lens of flexible learning and persistent stereotyped phases. This dynamic interaction characterizes the adaptive DM framework, which complements the frameworks in Figure 2. Each phase encompasses four modalities: motor, sensory, limbic, and cognitive, with each modality serving distinct functional roles, as shown in the corresponding boxes with smooth versus dashed red lines. The sensory modalities in both phases, such as auditory and visual, are coupled with different modulator profiles, including transfer ranges and persistence information.

Reflecting on the dynamic nature of cognitive processes in adaptive DM across various contexts, flexible learning, and persistent stereotyping can offer further insight (Figure 3). The interaction between these dual systems shapes the brain's competition circuit loops. These competitions, which encompass motor and nonmotor circuits, optimize their functioning through enhanced communication across four modalities: motor, limbic, sensory, and cognitive. The brain's dynamic resource allocation facilitates adaptability and efficient functioning in response to environmental demands. Consequently, adapting the dual systems is contingent upon these needs—adaptive DM flourishes on this balance, which can vary depending on the situation.

## 4 Discussion and Concluding Remarks

Kahneman's perspective on cognitive processes (Kahneman 2011), particularly his distinction between fast and slow thinking, has significantly influenced the field of human DM. Nevertheless, it encounters challenges within contemporary cognitive psychology. Sloman's 1996 research on DPT identified two types of reasoning: associative and rule‐based. He demonstrated that these systems complement each other, arguing that Kahneman's (2011) model oversimplifies cognitive processes. Researchers have critically examined the fast–slow division, stressed the complexity of cognitive processes, and advocated for acknowledging quantitative differences (Evans 2008; Evans and Stanovich 2013). Moreover, Newman, Gibb, and Thompson (2017) disputed existing assumptions regarding reasoning speed, suggesting that rule‐based reasoning can occur rapidly. Recent critiques from De Neys (2023) and Trommler and Hammann (2020) underlined this oversimplification; Lieder and Griffiths (2017) emphasized that DM is dynamic and contextually influenced. Therefore, it is indispensable to revisit DPT thoughtfully, considering ongoing dialogues within cognitive psychology. This approach can open new avenues for advancing research on adaptive DM.

Contextualizing Kahneman's (2011) work within the broader framework of DPT could offer a pragmatic solution. The ongoing debates about the validity of the System 1/System 2 dichotomy require a more integrated approach that considers the complexities and interdependencies of cognitive processes instead of relying on simplistic binary classifications. Explaining the neurobiological mechanisms underlying novelty‐seeking behavior can bridge the domains of foraging and general DM (i.e., JDM). Costa, Mitz, and Averbeck (2019) examined the subcortical substrates involved in explore–exploit decisions, revealing how neural circuits responsible for exploration influence DM across various contexts. Similarly, Menegas et al. (2018) elucidate the role of dopamine in approach‐retreat behaviors during foraging, which can be interpreted as a risk assessment process that may not entirely apply to traditional JDM tasks.

*Novelty* emerges as a fundamental factor in DM processes and should be integrated into this framework. It is necessary to distinguish between two forms of *novelty*: organismal and environmental. Organismal novelty refers to the intrinsic characteristics of an organism that may affect its DM processes. Conversely, environmental novelty refers to new or unfamiliar stimuli or contexts that an organism encounters; this *novelty* can stimulate exploration and shape DM strategies. An organism's phenotypic novelty—cognitive flexibility, openness to experience, and learning from past experiences—can impact how it interacts with the environment and adaptively processes information. Interestingly, the interplay between organismal and environmental novelty is reciprocal. An organism's capacity to adapt to or embrace *novelty* can influence how it perceives and interacts with its environment. *Novel* stimuli may trigger specific neural pathways associated with exploration and curiosity, as Jaegle, Mehrpour, and Rust (2019) discussed. Novelty‐seeking behaviors can drive exploration during sequential DM tasks, and it should be noted that *novelty* is not necessarily a surprise (Xu et al. 2021). Research by Laureiro‐Martínez and Brusoni (2018) emphasizes the significance of cognitive flexibility in adaptive DM, indicating that individuals with greater cognitive flexibility are more adept at navigating novel situations and making effective decisions.

While *novelty* is often used interchangeably with new information, it can imply a broader scope: unprecedented situations or stimuli that do not merely represent new information but require creative responses or problem‐solving. Thus, it could encompass complex scenarios that necessitate the development of new cognitive schemas. Our proposed framework offers an integrative perspective on adaptive DM, emphasizing the interplay of *novelty*, fast and slow thinking, and creative thinking (Figure 1). We elucidate the construction of adaptive DM in the context of creative thinking by incorporating the concepts of clustering and switching (Figure 2). Transitions between these modes reflect shifts between top‐down and bottom‐up approaches (Figure 2) and adequate communication between flexible learning and persistent stereotypical phases (Figure 3). We suggest explaining the temporal modulation and competition between motor and nonmotor loops to reveal how these circuits within their modalities interact during adaptive DM. Therefore, we advocate for empirical research on adaptive DM to prioritize the analysis of temporal dynamics within neural circuits. This approach, which emphasizes an integrated methodology, will support module definition, development, and evolution (Callebaut and Rasskin‐Gutman 2005).

Moreover, our framework advocates evaluating adaptability as the ability to respond to environmental changes in various contexts, such as personal, organizational, or societal. Consequently, it is essential to acknowledge the significance of transferable knowledge across diverse disciplines. Adaptation encompasses a variety of cognitive, emotional, and behavioral responses to new challenges or information, which resonates with Damasio's research on emotions that influence our sense of system‐level approaches (Damasio 1989) and self and consciousness (Damasio 1996). Damasio's pioneering concepts (1989, 1996) have questioned the traditional segregation between emotion and cognition, arguing that emotions are not merely distractions but are essential to achieving sociocultural homeostasis (Verweij and Damasio 2024). By integrating emotions into adaptive DM using bottom‐up and top‐down approaches, we can reclassify semantic search behaviors as fast or slow to capture the exemplary aspects of foraging behavior. The extensive research on foraging decisions provides valuable frameworks for understanding how organisms, including humans, make informed choices while searching for and utilizing knowledge and information. Through the lens of clustering and switching, the dynamic interplay between these two systems introduces further features in thought modes that influence adaptive DM. These features are marked in brain patterns of functional connectivity of creative ideation (Herault et al. 2024; Ovando‐Tellez et al. 2022, 2023; Ovando Tellez et al. 2024). Therefore, incorporating foraging theory can enrich its relevance to current cognitive psychology and behavioral economics discussions.

Another admittedly speculative approach suggests notable similarities between adaptive DM processes and concepts from behavioral ecology. In its original form, *Life history theory* explains the variations among species in allocating resources for somatic growth versus reproduction activity (Stearns 1992). When resources such as food and mates are scarce, species adapt by developing different life history strategies (LHS; Wang, Michalak, and Ackerman 2021). These strategies comprise various factors, including variances in growth rates, the age and size at which individuals reach sexual maturity, the number and size of offspring produced, mortality rates, maximum lifespan, and susceptibility to diseases (Stearns 1992). Initially, these differences were classified under the “r/K selection” (MacArthur and Wilson 1967). Species categorized as “r‐selected” (with “r” denoting growth rate) tend to be smaller in size, mature and reproduce early (often only once during their lifetime), produce a large number of offspring, provide minimal parental care, have relatively short lifespans, and exhibit higher vulnerability to diseases due to trade‐offs favoring reproduction over immune system strength. In contrast, “K‐selected” species (with “K” referring to carrying capacity) typically grow larger, reproduce multiple times throughout their lives, have fewer offspring, invest significantly in their offspring's survival, enjoy longer lifespans, and generally possess stronger immune systems compared to “r‐selected” species (Stearns 1992). A “fast” LHS (“r‐selected”) is often more profitable in uncertain and unpredictable environments. Meanwhile, a “slow” LHS (“K‐selected”) tends to be more reproductively successful in stable environments over the long term. Evolutionary psychologists have built upon earlier views—though these concepts are not universally accepted by biologists—suggesting that variations in LHS can be observed among individuals within the same species (Ellis et al. 2009, 2011). Ecological and genetic factors may influence whether an individual displays a relatively fast or slow LHS, which falls within the typical range for their species (Ellis et al. 2009, 2011). These environmental conditions often shape perceptions of current and future resource availability, affected by experiences during early developmental stages (Ellis et al. 2009). Encountering early adversity may lead an individual to adopt a faster LHS, characterized by earlier biological maturation, reduced emphasis on body maintenance, and limited parental care for offspring (Ellis et al. 2011). Variability in individual differences regarding cooperation, risk‐taking, interpersonal aggression, mating behaviors, executive functioning, and personality traits is partially shaped by early developmental conditions (Del Giudice 2014). Stability during early life is associated with higher levels of conscientiousness and agreeableness in personality traits. Conversely, individuals who endure harsh parenting, exposure to violence, or poverty tend to exhibit the opposite outcomes. These individuals tend to mature earlier, engage in sexual activities at a younger age, and show increased risk‐taking behaviors along with diminished inhibitory control (Belsky, Steinberg, and Draper 1991; Ellis et al. 2011). A relatively “faster” LHS correlates with higher scores in extraversion, openness to experience, and neuroticism (Del Giudice 2014).

Before this background (considerably abridged for space and comprehensiveness), we propose to extend our framework further and argue that adaptive DM follows a similar logic within individuals. Our framework also extends to understanding animal behavior, which we propose to exist on a continuum with human behavior. Ant colonies exhibit varying exploratory and exploitative behaviors based on levels of *uncertainty*, responding at dissimilar speeds—either quickly or slowly (Blight et al. 2016). As cultural beings, humans have developed a vast array of “memes” (Dawkins 1976) that are transmitted culturally. Many memes can endure beyond immediate decisions, whether communicated orally or in written formats. Hence, analyzing the behavior of adaptive DM in animals can provide valuable perspicuity. However, it remains problematic to operationalize the distinction between fast and slow behaviors in animals, a task that is already complex when applied to humans. While the evolutionary perspective on adaptive DM offers valuable insights, operationalizing this distinction across species poses significant challenges. One of them is applying the two systems to animals, which involves variability in cognitive complexity across different species.

The following anecdote may illustrate our point. One of us (M.B.) recently visited the Museum of Prehistory in Blaubeuren, Southern Germany. This museum showcases copies and originals of stone‐age artifacts, including the renowned “Lion Man” and other figurines dating back 40,000 years. Among the most remarkable artistic creations are several flutes, one crafted from a mammoth tusk. This particular flute is one of the oldest musical instruments ever discovered. A modern replica made by Hein (2021) allows us to experience the deep, warm sound of the flute. It resembles the pentatonic scale similar to the compositions of Ennio Morricone—this was M.B.'s impression. Creating such a delicate musical instrument demands many hours of meticulous work, often dozens or more. Accurately drilling the finger holes at the correct intervals requires a profound knowledge of the flute's physics. In other words, such skillful craftsmanship could not have developed in an environment solely marked by harshness, constant danger, and famine. Instead, manufacturing this delicate instrument suggests the presence of periods of relaxation, social gatherings, and prospects for emotional connection through early musical expressions. Consequently, it is imperative to acknowledge the limitations of drawing parallels between the behavioral ecological perspective and creative thought, and whether thinking fast or slow has ever paid off reproductively, which is unknown.

In summary, while emphasizing *novelty* in foraging decisions holds substantial value, clarifying how this knowledge can be applied to the contexts of adaptive DM is essential. By focusing on the explore–exploit trade‐off and integrating neurobiological perspectives, we can examine the role of *novelty* in foraging underlying adaptive DM scenarios. Although the evolutionary framework for adaptive DM could serve as a valuable tool for comprehending this process across various species, applying two systems to animals must be cautiously approached and warrants further discussion. Therefore, researchers should consider constructing more sophisticated models that accurately echo the adaptive DM strategies among different species.

## Author Contributions

**Radwa Khalil:** conceptualization, investigation, resources, visualization, writing – original draft, writing – review and editing. **Martin Brüne:** conceptualization, investigation, writing – original draft, writing – review and editing.

## Ethics Statement

The authors have nothing to report.

## Conflicts of Interest

The authors declare no conflicts of interest.

### Peer Review

The peer review history for this article is available at https://www.webofscience.com/api/gateway/wos/peer‐review/10.1111/ejn.70024.

## Data Availability

The authors have nothing to report.

## Endnotes

^1^
Acquiring further information diminishes uncertainty, though it may simultaneously expose additional potential risks. Consequently, an inverse relationship exists between the quantity of information gathered and the level of uncertainty. This uncertainty results from the decision‐maker's limited knowledge, which can emerge at any point in the DM process (Dow 2012).

^2^
Brunswik posited that psychology should prioritize statistical descriptions of adaptive behavior (Brunswik 1943). Rather than isolating a limited number of independent variables for manipulation in controlled experiments and examining their effects on a dependent variable, he argued that psychological research should investigate how organisms adapt to their environments. He termed this approach the lens model (Brunswik 1955), a theoretical framework for understanding how individuals judge based on environmental cues. Consequently, Brunswik advocated for using a representative design in psychological experiments to preserve the causal structure relevant to the real‐world context of organisms. The notions of representative design and the lens model emphasize the limitations of human cognition when engaging with complex ecological systems (Kozyreva and Hertwig 2021).

^3^
The research endeavors of Gigerenzer's and Hertwig's teams at the Max Planck Institute for Human Development are highly influential. They investigate the algorithmic structure of simple heuristics and the adaptive psychological mechanisms that explain their adoption and effectiveness. Both groups operate under the premise that the expected utility theory is not a suitable basis for a descriptive theory of JDM.

^4^
Gigerenzer's system prominently criticizes the roles that optimization, rationality, and consistency play in the conventional normative standards for human rationality (Gigerenzer and Brighton 2009). Gigerenzer (1996) particularly challenges how these standards are reflected in Kahneman and Tversky's perspective on heuristics and biases.

^5^
Neophobia is characterized as an aversion to novel cues. Greenberg (1984) pioneered the study of neophobic responses in bird foraging decisions, while neophilia, or the attraction to novelty, can drive innovation (Mettke‐Hofmann, Winkler, and Leisler 2002).

^6^
Small samples amplify correlations, making them easier to detect (Kareev 1995).

^7^
Sensory modalities refer to sensory channels through which we perceive and gather information from our surroundings (Munoz and Blumstein 2012).

^8^
It refers to the cognitive processes in seeking and processing information relevant to DM to optimize resource acquisition while minimizing costs.

^9^
This theory clarifies why individuals prioritize information that is readily accessible or perceived as most valuable. While novelty is a crucial factor in foraging behavior—where seeking new resources is a vital part of the DM process—it may not be as easily applied to typical experimental situations in JDM, such as lotteries or risk assessments, which often do not include an explicit novelty component.

^10^
Available definitions of biological novelty include traits resulting from development differences, enabling new functions, fitness peaks, and underlying adaptive radiation (Pigliucci 2008; Hallgrímsson et al. 2012).
